# A Clinical Study of Functional Disorders of the Heart

**Published:** 1918-09

**Authors:** 


### MEDICINE

***A Clinical Study of some Functional Disorders of the Heart Which Occur in Soldiers.*** By J. E. MAC ILWAINE, Former Physician in St. John Ambulance Brigade Hospital, B. E. F. *Journal of the Royal Army Medical Corps*, April, 1918.

The term " functional disorder " is one which is vague and wide, but for lack of a better it is to be preferred to the still more indefinite classification used in Army work, namely D. A. H. (Disordered Action of the Heart). The cases which are considered in this article have been described as functional because clinical examination failed to reveal any organic disease which could be held responsible for the evidences of cardiac disturbance.

One examination rarely proves adequate for the decision as to the disposal of the soldier, and it has been found necessary to use what is known as an " effort test ", which consists of submitting the cases of doubtful pathogenesis to a six-weeks course of graduated physical drill. Roughly speaking, about one-half of the number treated were classifield as fit for active service.

The picture of the affection varies, but the important factor is the failure of the soldier to endure a moderate amount of effort or even a small degree of emotion, without collapsing or fainting. The histories given by the men are the same in substance, but vary as to the principal or predominant feature, i. e., pain, dyspnea, palpitation, vertigo, weakness, fainting, exhaustion, etc. A soldier will also complain of these symptoms occurring just as he is going to sleep, or else he will wake with the pain and palpitation and choking sensation.

In cases of this affection it was noticeable that if, for instance, a man did fall out on a hill, he was often able after a short rest to follow on and finish the march. The feature of these men after falling out has been their good color, hurried respiration, profuse sweating and tachycardia. The condition is so easy to recognize that the most one can do is to find out by clinical examination whether there is associated with it any organic disease.

To begin with all the men were considered as possible subjects of hyperthyroidism, unless that possibility was unsupported by examination. The condition of the tonsils and teeth was noted in each man. Efforts were directed to determine whether there was any organic heart disease present, endocardial or myocardial. It was found in many cases that the syndrome was associated with definite organic changes in the respiratory or cardio-vascular systems, in a few cases with Grave's disease, and in some that the man was still suffering from pyrexia of uncertain origin.

The state of the nervous system as indicated by the reflexes would warrant the description of cardiac neurasthenics in two-thirds of the cases. It was strongly felt that the neurasthenic state had to be considered an important factor in forming a prognosis.

The writer delares that the etiology of the syndrome of " Irritable Heart ", " Soldier's Heart ", " Cardiac irritability " may be said to be emotion and intoxication, which two factors bring about a lowered threshold of stimulation in the involuntary neuro-muscular system. Then there is the individual response to these two factors to be considered in addition, an exaggeration of which may, under apparently healthy conditions, bring about the breakdown of the mechanism opposed to it.

*Treatment.* The author states that it was found impossible from a practical point of view that these men could be cured sufficiently for active service by hospital treatment, and it was then decided to try treatment by graduated exercise on the scheme which had been used at the Military Hospital at Hampstead. Consequently all suitable D. A. H. cases were sent to the convalescent depot, where excellent results were obtained by carefully maintaining a pleasant social-athletic spirit.

*Prognosis.* The problem of dealing with these men in the army is a simple one compared to the question of their treatment when the army has discarded them and they return to civil life.

